# Ocular manifestations of obstructive sleep apnea: a systematic review and meta-analysis

**DOI:** 10.1007/s00417-023-06103-3

**Published:** 2023-05-25

**Authors:** Gabriella Bulloch, Ishith Seth, Zhuoting Zhu, Sharanya Sukumar, Alan McNab

**Affiliations:** 1 Department of Ophthalmology and Surgery, Centre for Eye Research Australia, Victoria, 3002 Australia; 2 Central Clinical School, Monash University, Melbourne, 3004 Australia

**Keywords:** Obstructive sleep apnea, Ocular comorbidities, Floppy eyelid syndrome, Keratoconus

## Abstract

**Background:**

The association of obstructive sleep apnea (OSA) with development of eye diseases is unclear. This current systematic review and meta-analysis attempts to summarize and analyze associations between OSA and ocular disorders in the literature.

**Methods:**

PubMed, EMBASE, Google Scholar, Web Of Science, and Scopus databases were searched from 1901 to July 2022 in accordance with the Preferred Reporting in Systematic Review & Meta-Analysis (PRISMA). Our primary outcome assessed the association between OSA and the odds of developing floppy eyelid syndrome (FES), glaucoma, non-arteritic anterior ischemic optic neuropathy (NAION), retinal vein occlusion (RVO), keratoconus (KC), idiopathic intracranial hypertension (IIH), age-related macular degeneration (AMD), and central serous chorioretinopathy (CSR) through odds ratio calculated at the 95% confidence interval.

**Results:**

Forty-nine studies were included for systematic review and meta-analysis. The pooled OR estimate was highest for NAION [3.98 (95% CI 2.38, 6.66)], followed by FES [3.68 (95% CI 2.18, 6.20)], RVO [2.71(95% CI 1.83, 4.00)], CSR [2.28 (95% CI 0.65, 7.97)], KC [1.87 (95% CI 1.16, 2.99)], glaucoma [1.49 (95% CI 1.16, 1.91)], IIH [1.29 (95% CI 0.33, 5.01)], and AMD [0.92 [95% CI 0.24, 3.58] All observed associations were significant (*p* < 0.001) aside from IIH and AMD.

**Conclusion:**

OSA is significantly associated with NAION, FES, RVO, CSR, KC, and glaucoma. Clinicians should be informed of these associations so early recognition, diagnosis, and treatment of eye disorders can be addressed in at-risk groups, and early referral to ophthalmic services is made to prevent vision disturbances. Similarly, ophthalmologists seeing patients with any of these conditions should consider screening and referring patients for assessment of possible OSA. 
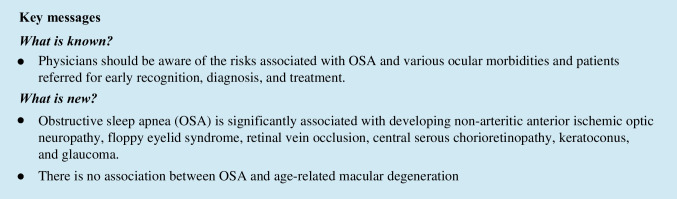

## Introduction

Obstructive sleep apnea (OSA) is a common sleep disorder with disturbed breathing resulting from repetitive total or partial collapse of the upper respiratory tract during sleep. Breathing cessation generally lasts for 10 to 30 s causing swings between hypoxia and reperfusion, which if chronic, ultimately leads to pulmonary hypertension [1]. As the most common sleep disorder, its prevalence ranges from 22 to 24% in men, 9 to 17% in women, and 6% in adolescents. OSA is now considered a global public health issue as incidence climbs and its associated risks with other diseases and mortality grow [2]. OSA is a well-known major risk factor for many cardiovascular and metabolic health issues [3], but is also significantly associated with ophthalmic disorders including floppy eyelid syndrome (FES) [4–9], papilledema [10], non-arteritic anterior ischemic optic neuropathy (NAION), [11–20] keratoconus (KC), [21–25] central serous chorioretinopathy (CSR) [26–29], retinal vein occlusion (RVO) [30–33], glaucoma [5, 34–52], age-related macular degeneration (AMD) [51, 52], and idiopathic intracranial hypertension (IIH) [14, 53–55]. Many hypotheses for these associations have been proposed, but intermittent hypoxia, excessive sympathetic stimulation, oxidative stress, and damaging effects of endothelin-1 are mechanisms thought to be involved with the pathophysiology [56].

These ocular morbidities associated with OSA have resulted in research to better understand underlying mechanisms, specific populations at risk, and prediction of morbidity. A better understanding surrounding these could lead to referral of at-risk groups to ophthalmic services shortly after diagnosis to preserve sight and prevent significant morbidity, and conversely, screening and referral pathways for ophthalmologists may be established for patients at-risk for OSA with common ocular manifestations.

Before these interventions can be widely acknowledged as critical for preserving eye health, a consensus about the associations of ocular disorders with OSA needs to be met. One systematic review and meta-analysis in the past has been conducted; however, an influx of new studies and the exclusion of other potentially important conditions like KC and IIH leaves room for conjecture [57]. This systematic review and meta-analysis attempts to assist in this process by summarizing and analyzing associations between OSA and ocular disorders in the current literature.

## Methods

### Methodology statement

This review adhered to the Preferred Reporting in Systematic Review & Meta-Analysis (PRISMA) [58] guidelines and was listed on the PROSPERO, International Prospective Register of Systematic Review (CRD42022314672). There were no study restrictions imposed on different populations, races, ethnicity, origin, and language.

### Literature search

Online electronic databases (PubMed, EMBASE, Google Scholar, Web Of Science, and Scopus) were searched till July 2022 using the search terms: (“Obstructive sleep apnea/hypopnea syndrome” or “OSAHS” or “sleep apnea syndrome” or “OSA” or “obstructive sleep apnea”) and the ophthalmologic disorders individually (“floppy eyelid syndrome,” “glaucoma,” “non-arteritic anterior ischemic optic neuropathy,” “retinal vein occlusion,” “keratoconus,” “idiopathic intracranial hypertension,” “age related macular degeneration,” “central serous chorioretinopathy”). The search terms were used in different combinations. No age, gender, and population filters were imposed. Manual search of included studies and previous reviews’ references was performed.

### Inclusion and exclusion criteria

The established inclusion criteria were as follows: (1) all published studies evaluating the association between OSA and association of specific ophthalmologic disorders, (2) all full-text studies including randomized control trials, original research articles, descriptive and analytic studies (cohort or case-control), (3) studies providing sufficient data to calculate odds ratio (OR) and 95% confidence interval (CI), and (4) studies published in English and conducted on human participants. There was no limit on the population group in terms of age, sex, ethnicity, or co-morbidities.

The exclusion criteria were as follows: (1) studies not measuring conclusive risk estimates, (2) studies without available full-text, (3) poster or scientific presentations, (4) reviews, meta-analysis, opinion articles, surveys, letter to editor, short communications, case reports, case series, abstracts, commentaries, and book chapters.

### Outcome measures

The primary outcome measures assessed the strength of association between OSA and associated ophthalmologic comorbidities like floppy eyelid syndrome, glaucoma, non-arteritic anterior ischemic optic neuropathy, retinal vein occlusion, keratoconus, idiopathic intracranial hypertension, age-related macular degeneration, and central serous chorioretinopathy, in terms of odds ratio calculated at 95% confidence interval (CI).

### Data extraction and quality assessment

Two authors independently (IS and GB) screened all titles and abstracts against predefined inclusion and exclusion criteria, and any disparity in either selecting eligible studies or assessing findings between the two reviewers was resolved through consultation with a third reviewer (SS). The complete texts of the studies were then obtained and read in full to fulfill the final inclusion. Any differences in articles selected by the two were discussed to reach a decision regarding inclusion. The reference lists of screened articles were also reviewed for any missed literature. The information recorded for each selected study included the name of the first author, publication year, study design, participant selection, total number of cases and controls, methods for the diagnosis of OSA, systemic disease prevalence (hypertension, diabetes, and body mass index), presence of adjustment for covariates, CPAP status, and the author’s remarks/conclusions.

### Statistical analysis

The meta-analysis was performed using review manager (RevMan version 5.4, Cochrane collaboration, Oxford, UK). Summary of OR estimates from each study was calculated by a random-effects Mantel- Haenszel method, and results presented as an odds ratio (OR) with 95% confidence intervals (CIs). Statistical difference between the groups was considered to be present if the pooled 95% CI did not include 1 for the respective OR. For each factor analyzed, a forest plot showing the respective odds ratios or standardized mean differences with their corresponding 95% confidence interval for each study and the pooled data were generated. The test of overall effect was assessed using the *Z* statistics on RevMan v5.4 with statistical significance set at *p* < 0.05.

Heterogeneity (inconsistency) between studies was evaluated using the Cochrane *Q* (Chi^2^ test) and I^2^ statistics in RevMan v5.4. Estimates of degree of heterogeneity using *I*^2^ were made by setting 25%, 50%, or 75% as limits for low, moderate, or high heterogeneity, respectively. Random-effects model with weighting of the studies was used when there was heterogeneity between studies with *I*^2^ values of over 50%. All *p* values were two-tailed and considered statistically significant if less than 0.05.

## Results

### Study selection

**Fig. 1:**
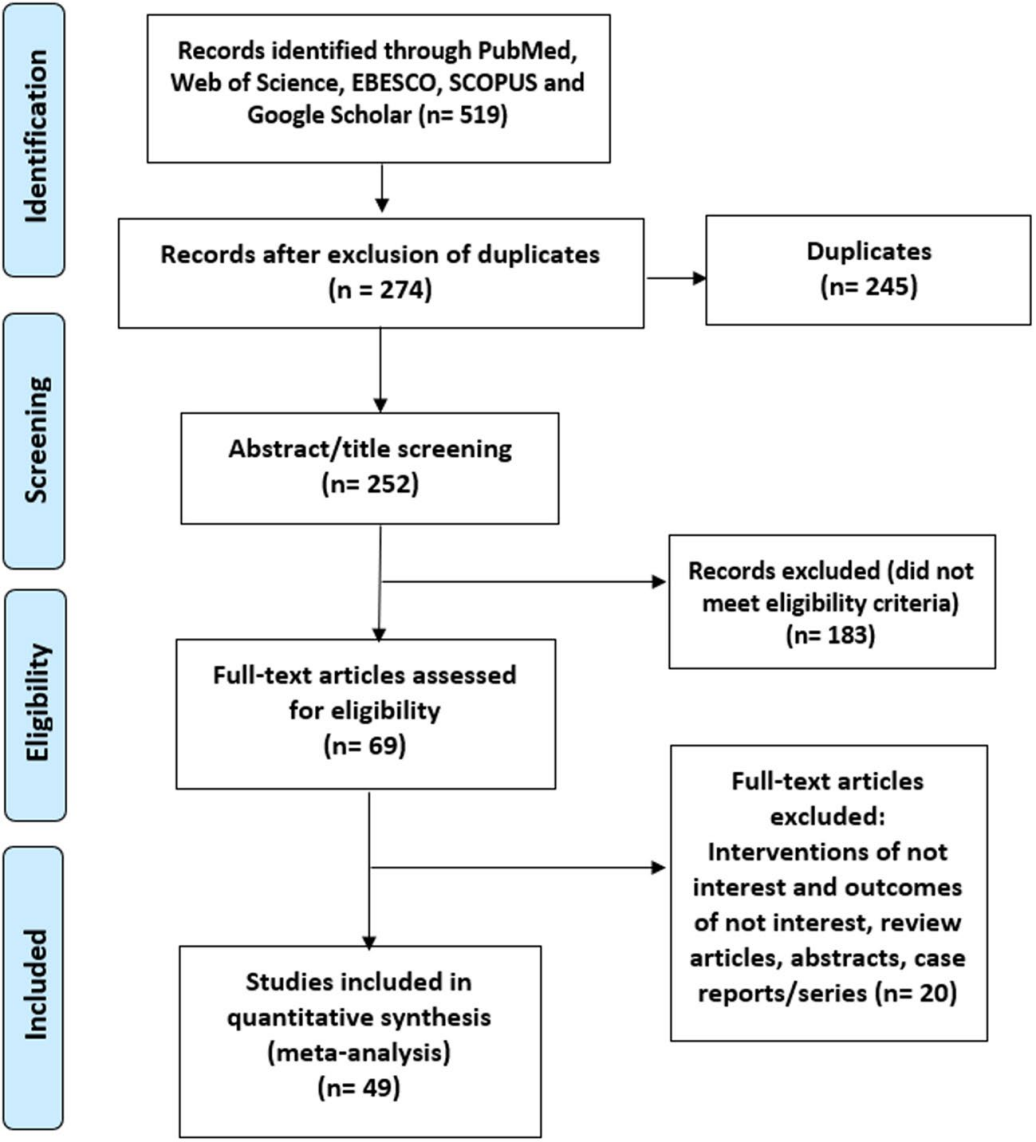
Summary of study selection process Preferred Reporting Items for Systematic Review and Meta-Analyses flow diagram

A total of 519 potentially eligible records were extracted in the initial data retrieval process. During the screening, 245 records were omitted following duplication screening, and 22 were eliminated for not being in English. Of the 252 records assessed for eligibility, 183 studies were excluded for not meeting the inclusion criteria. Forty-nine studies were finally included for quantitative assessment and meta-analysis. Mild to moderate heterogeneity of included studies was observed based on *I*^2^ statistics less than 50%. Characteristics of the included studies are detailed in Table 1. The process used to search and identify studies is illustrated in Fig. 1. Basic cohort characteristics are listed in Table 2. The average cohort percentages for diabetes and hypertension were 24.04% and 38.12%, respectively, from 11 and nine studies reporting on these statistics in OSA patients. The average BMI was 30.4, reported from 13 studies. Just two studies reported their participants using CPAP, 30 studies identified diagnosis of OSA at the time of the study and were deemed CPAP-naive, while the remaining 17 used retrospective data and reported no use of CPAP.

**Table 1:** Characteristics of included studies

Authors	Year of study	Study design	Region/country	Ocular condition	Population
					Cases/controls
Onen et al. [34]	2000	Case control	France	Glaucoma	212/218
Marcus et al. [35]	2001	Retrospective chart order review	Georgia	Glaucoma	37/30
Mojon et al. [11]	2002	Case control	Switzerland	NAION	17/17
Tsang et al. [36]	2006	Case control	China	Glaucoma	41/35
Girkin et al. [37]	2006	Nested case control	USA	Glaucoma	667/6667
Karger et al. [4]	2006	Cross-sectional	USA	FES	44/15
Palombi et al. [12]	2006	Case control	France	NAION	27/5615
Sergi et al. [38]	2007	Case control	Italy	Glaucoma	51/40
Li et al. [13]	2007	Matched case control	USA	NAION	73/73
Boonyaleephan et al. [39]	2008	Cross-sectional	Thailand	Glaucoma	44/42
Roberts et al. [40]	2009	Case control	Australia	Glaucoma	52/60
Kadyan et al. [5]	2010	Case control	UK	Glaucoma	89/26
				FES	89/26
Ezra et al. [6]	2010	Case control	UK	FES	102/102
Fraser et al. [53]	2010	Case control	UK	IIH	24/48
Boyle Walker et al. [41]	2011	Retrospective record review	USA	Glaucoma	2725/68236
Lin et al. [42]	2011	Cross-sectional	Taiwan	Glaucoma	209/38
Nowak et al. [43]	2011	Semi prospective, cross-sectional	Poland	Glaucoma	34/18
Stein et al. [14]	2011	Database study	USA	Glaucoma	151633/2030682
				IIH	156332/2102011
				NAION	156336/2102725
Chou KT et al. [30]	2012	Matched control cohort	Taiwan	RVO	5965/29669
Saidel MA et al. [21]	2012	Case control	USA	Keratoconus	92/92
Beis et al. [7]	2012	Case control	Greece	FES	81/54
Chambe et al. [8]	2012	Cross-sectional	France	FES	89/38
Lin et al. [44]	2013	Cohort	Taiwan	Glaucoma	1012/5060
Moghimi et al. [45]	2013	Cross-sectional	Iran	Glaucoma	51/54
Khandgave et al. [46]	2013	Cross-sectional	India	Glaucoma	40/40
Arda et al. [15]	2013	Case control	Turkey	NAION	20/20
Bilgin et al. [16]	2013	Case control	USA	NAION	27/27
Pihlblad and Schaefer [22]	2013	Case control	USA	Keratoconus	15/15
Acar et al. [9]	2013	Cross-sectional	Turkey	FES	254/26
Aptel et al. [47]	2014	Prospective cohort	France	Glaucoma	6754/2826
Muneisa et al. [48]	2014	Cross-sectional	Spain	Glaucoma	127/25
Bilgin et al. [49]	2014	Case control	Turkey	Glaucoma	24/24
Gencer et al. [23]	2014	Prospective case control	Turkey	Keratoconus	146/146
Bandi MFG et al. [17]	2015	Case control	Iran	NAION	19/31
NadeeranM et al. [24]	2015	Prospective case control	Iran	Keratoconus	616/616
Woodward et al. [25]	2016	Database	USA	Keratoconus	16053/16053
Cestari et al. [18]	2016	Retrospective cohort study	USA	NAION	977/1380500
Chatziralli I et al. [28]	2017	Case control	Greece	CSR	183/183
Keenan TDL et al. [51]	2017	Record-linkage study	UK	AMD	67786/2684131
Agard et al. [31]	2018	Case control	France	RVO	69/45
Bagabas et al. [50]	2019	Case control	Saudi Arabia	Glaucoma	45/39
Liu PK et al. [29]	2019	Nationwide population-based study	Taiwan	CSR	10753/322590
Wang YH et al. [32]	2019	Case-control study	China	RVO	30/30
Ardissino et al. [54]	2019	Database study	UK	IIH	607/230792
Radojicic et al. [55]	2019	Prospective cohort	Serbia	IIH	219/67
Sun et al. [19]	2019	Retrospective cohort study	Taiwan	NAION	8488/33952
Yang et al. [20]	2019	Retrospective cohort study	Republic of Korea	NAION	919/9190
Wan W et al. [33]	2021	Case-control study	China	RVO	45/45
Han X et al. [52]	2021	Prospective cohort	UK	AMD	9182/493231

**Table 2:** Cohort characteristics of included studies

Authors	Characteristics of OSA participants					
	Eye disease studied	DM (%)	HTN (%)	BMI (mean)	CPAP status (%)	Model adjustment*
Onen et al. [34]	Glaucoma	NR	NR	NR	Untreated	None
Marcus et al. [35]	Glaucoma	45.9	51.4%	30.2	Untreated	None
Mojon et al. [11]	NAION	8	33.3	26.7	Untreated	None
Tsang et al. [36]	Glaucoma	NR	NR	29.5	Untreated	BMI
Girkin et al. [37]	Glaucoma	NR	NR	NR	NR	DM, HTN etc
Karger et al. [4]	FES	NR	NR	NR	Untreated	BMI etc
Palombi et al. [12]	NAION	NR	NR	12.5% (> 30 BMI)	Untreated	None
Sergi et al. [38]	Glaucoma	NR	NR	NR	Untreated	None
Li et al. [13]	NAION	29	60	NR	Untreated	DM, HTN, BMI etc
Boonyaleephan et al. [39]	Glaucoma	NR	NR	28.17	Untreated	None
Roberts et al. [40]	Glaucoma	NR	NR	NR	Untreated	None
Kadyan et al. [5]	Glaucoma	NR	NR	NR	100	BMI etc
	FES	NR	NR	NR	82.14	
Ezra et al. [6]	FES	NR	NR	NR	Untreated	None
Fraser et al. [53]	IIH	NR	NR	NR	NR	BMI etc
Boyle Walker et al. [41]	Glaucoma	NR	NR	NR	NR	None
Lin et al. [42]	Glaucoma	NR	NR	26.4	Untreated	None
Nowak et al. [43]	Glaucoma	NR	NR	31.5	Untreated	None
Stein et al. [14]	Glaucoma	NR	NR	NR	NR	DM, HTN, BMI > 30, etc
	IIH	NR	NR	NR	NR	
	NAION	NR	NR	NR	NR	
Chou KT et al. [30]	RVO	17.85	33.93	NR	NR	DM, HTN etc
Saidel MA et al. [21]	Keratoconus	NR	NR	33.9	NR	BMI etc
Beis et al. [7]	FES	Excluded	NR	33.5	Untreated	Stratified by BMI group
Chambe et al. [8]	FES	NR	NR	NR	Untreated	BMI etc
Lin et al. [44]	Glaucoma	50.5	22.3	3.3% (over BMI 30)	NR	DM, HTN, BMI etc
Moghimi et al. [45]	Glaucoma	NR	NR	NR	NR	None
Khandgave et al. [46]	Glaucoma	0%	10%	NR	`Untreated	None
Arda et al. [15]	NAION	NR	NR	NR	Untreated	None
Bilgin et al. [16]	NAION	NR	NR	NR	Untreated	None
Pihlblad and Schaefer [22]	Keratoconus	NR	NR	NR	Untreated	None
Acar et al. [9]	FES	NR	NR	NR	Untreated	None
Aptel et al. [47]	Glaucoma	20.74	NR	30.65	NR	BMI, HTN etc
Muneisa et al. [48]	Glaucoma	NR	NR	NR	Untreated	BMI
Bilgin et al. [49]	Glaucoma	25	58.3	26.8	Untreated	None
Gencer et al. [23]	Keratoconus	NR	NR	31	Untreated	BMI etc
Bandi MFG et al. [17]	NAION	NR	NR	NR	Untreated	Backward step-wise logistic regression
NadeeranM et al. [24]	Keratoconus	NR	NR	29.86	Untreated	BMI etc
Woodward et al. [25]	Keratoconus	NR	NR	NR	NR	DM etc
Cestari et al. [18]	NAION	NR	NR	NR	NR	None
Chatziralli I et al. [28]	CSR	NR	NR	NR	NR	Backward step-wise logistic regression
Keenan TDL et al. [51]	Glaucoma	NR	NR	NR	Untreated	Etc
Agard et al. [31]	RVO	NR	NR	NR	Untreated	DM, HTN, BMI etc
Bagabas et al. [50]	Glaucoma	44	71	37.4	Untreated	DM, HTN, BMI etc
Liu PK et al. [29]	CSR	NR	NR	NR	44.67	DM, HTN etc
Wang YH et al. [32]	RVO	NR	NR	NR	NR	DM, HTN, BMI etc
Ardissino et al. [54]	IIH	NR	NR	NR	NR	None
Radojicic et al. [55]	IIH	NR	NR	NR	NR	None
Sun et al. [19]	NAION	6.33	2.84	NR	NR	HTN, DM etc
Yang et al. [20]	NAION	NR	NR	NR	NR	HTN, DM, etc
Wan W et al. [33]	RVO	NR	NR	NR	Untreated	BMI etc
Han X et al. [52]	AMD	17.2	NR	79.9 (> 30 BMI)	NR	SBP, DM etc
Average across studies	NA	24.04	38.12	30.4	NA	NA

^*^For hypertension, diabeters, and body mass index, these were summarized as HTN, DM, and BMI, respectively, etc., indicates other variables were adjusted for

### Glaucoma

**Fig. 2:**
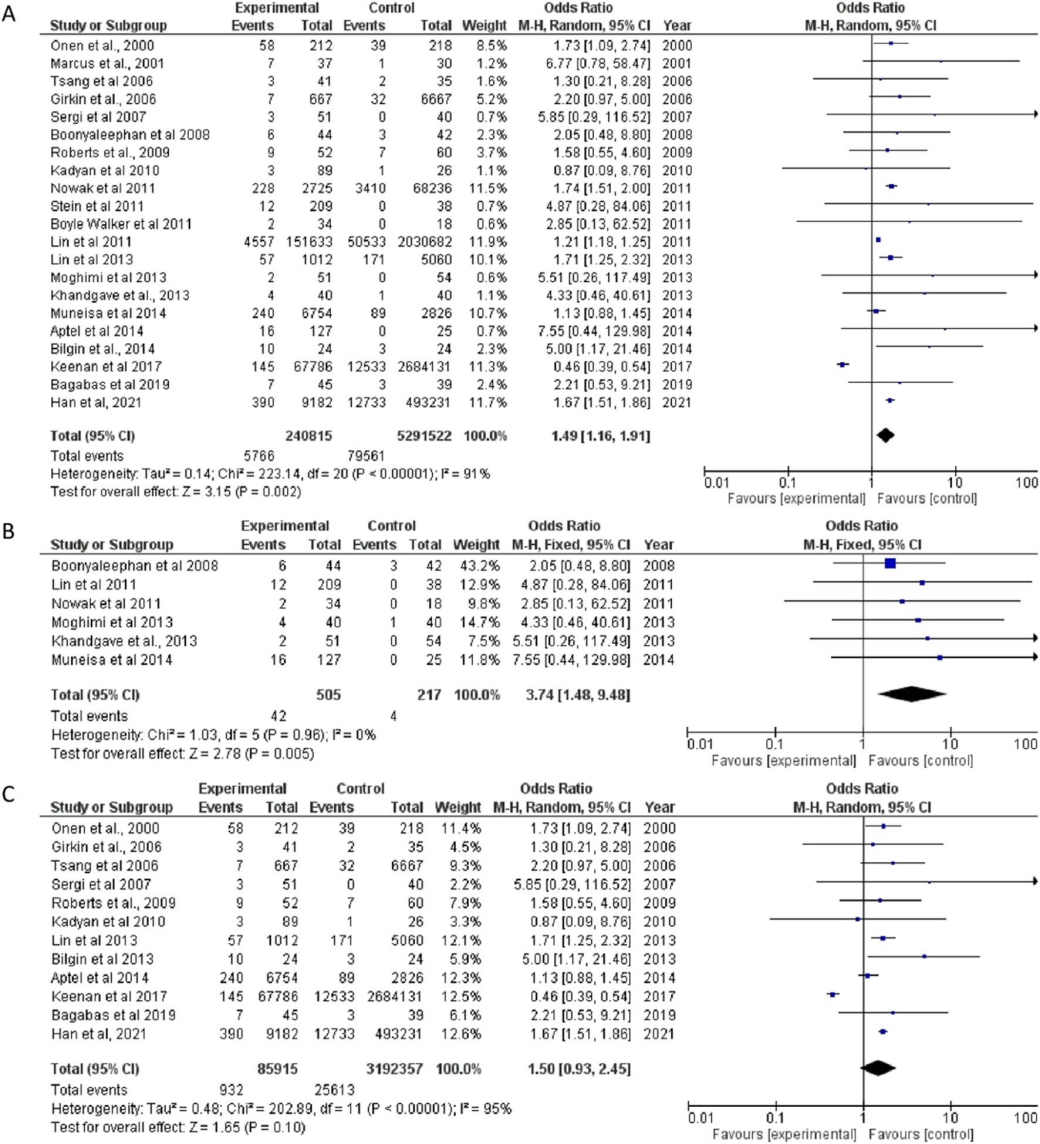
Forest plot showing odds of glaucoma in OSA patients in (**A**) all included studies; (**B**) cross-sectional studies; and (**C**) prospective studies

A total of 21 studies were included (8 case control, 6 cross-sectional, 4 cohort, 2 retrospective chart order review, and 1 database study). The overall pooled OR estimate was assessed as 1.49 (95% CI 1.16, 1.91). For cross-sectional studies, the aggregate OR estimate was 3.74 (95% CI 1.48, 9.48), much higher compared to prospective studies where pooled OR estimate was 1.50 (95% CI 0.93, 2.45) (Fig. 2).

### Floppy eyelid syndrome

**Fig. 3:**
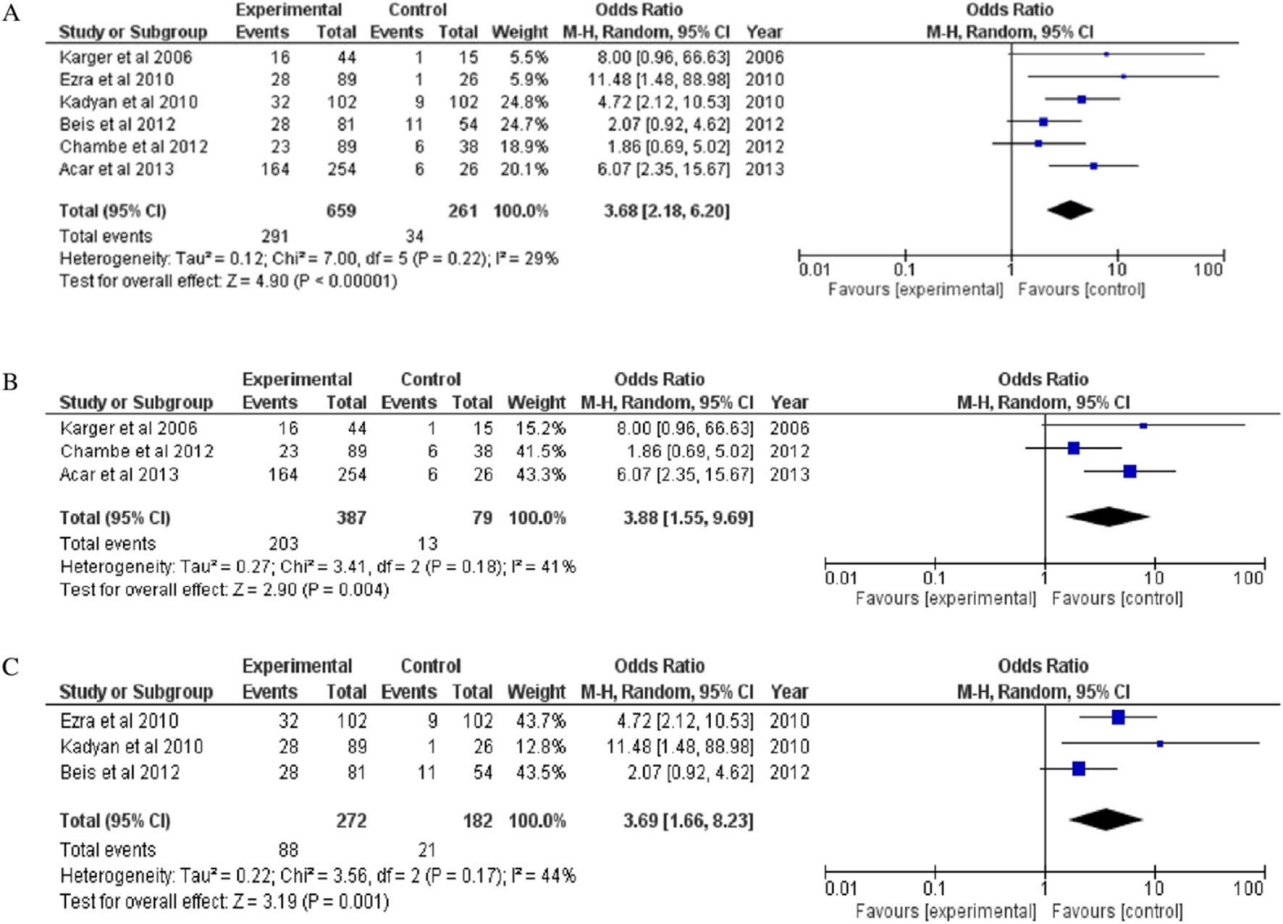
Forest plot showing odds of FES in OSA patients in (**A**) all included studies; (**B**) cross-sectional studies; and (**C**) prospective studies

Of the six studies included for analysis, three were cross-sectional studies and three case-control studies. While the overall pooled OR was 3.68 (95% CI 2.18, 6.20), the aggregate OR estimate for cross-sectional studies was 3.88 (95% CI 1.55, 9.69) and prospective studies was 3.69 (95% CI 1.66, 8.23) (Fig. 3).

### Central serous chorioretinopathy

**Fig. 4:**
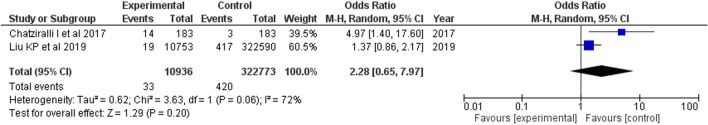
Forest plot showing odds of CSR in OSA patients

One case-control study and one population-based study were included for analysis. The pooled OR was assessed to be 2.28 (95% CI 0.65, 7.97) (Fig. 4).

### Keratoconus

**Fig. 5:**
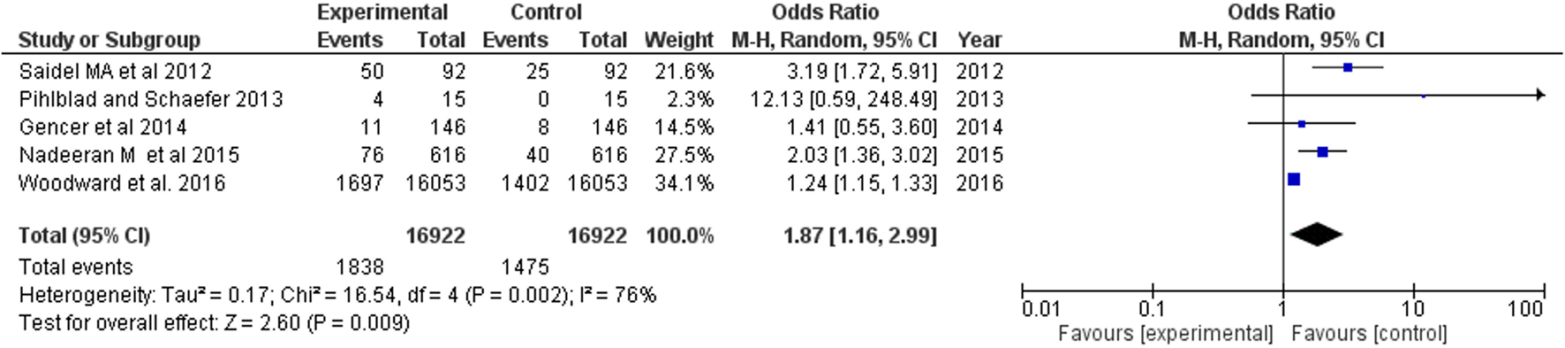
Forest plot showing odds of keratoconus in OSA patients

The study included four case-control studies and one database study reporting OSA incidence in keratoconus patient population. A total of 1838 OSA cases were noted among 16,922 pooled keratoconus cases, with pooled OR estimate analyzed as 1.87 (95% CI 1.16, 2.99) (Fig. 5).

### Non-arteritic anterior ischemic optic neuropathy

**Fig. 6:**
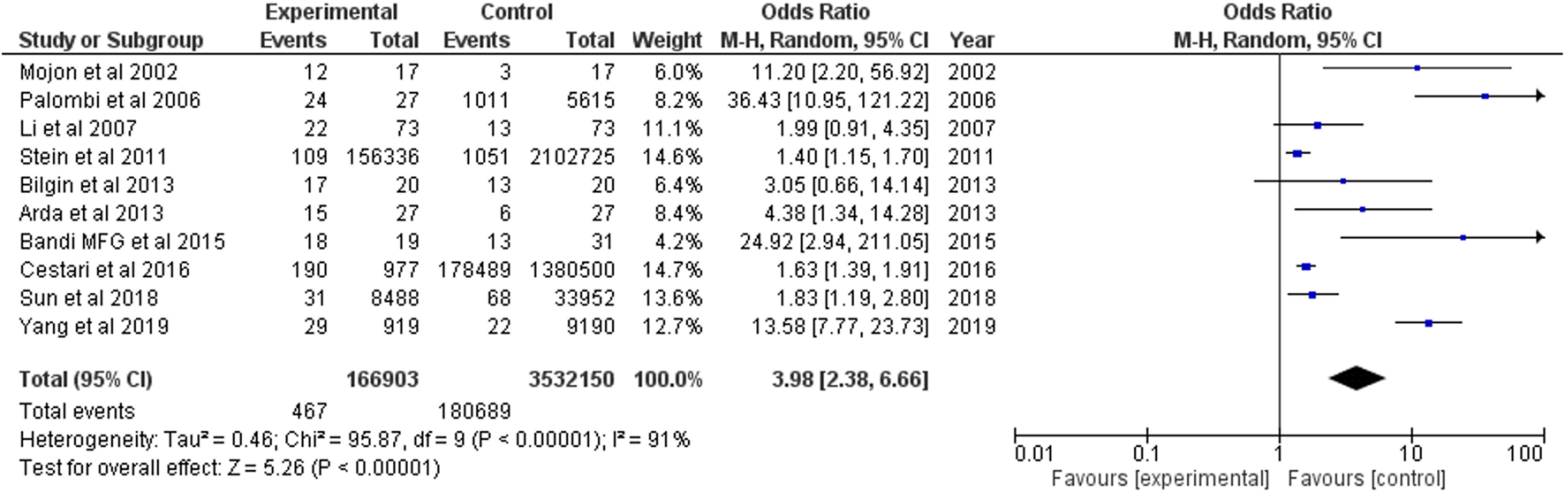
Forest plot showing odds of NAION in OSA patients

A total of ten studies were included, of which six were case-control studies, three were retrospective cohort studies, and one database study which reported risk of OSA in NAION population. Pooled OR estimates observed was 3.98 (95% CI 2.38, 6.66) (Fig. 6).

### Retinal vein occlusion

**Fig. 7:**
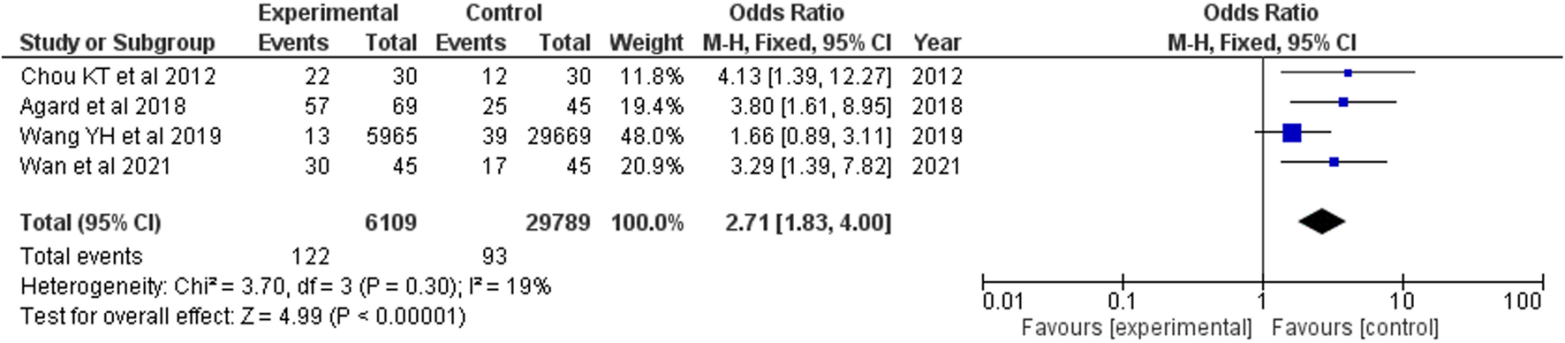
Forest plot showing odds of RVO in OSA patients

Of the four studies included, three were case-control studies reporting prevalence of OSA in RVO cases, and one was a population-based study reporting prevalence of RVO in OSA group. A pooled OR of 2.71 (95% CI 1.83, 4.00) was analyzed (Fig. 7).

### Idiopathic intracranial hypertension

**Fig. 8:**
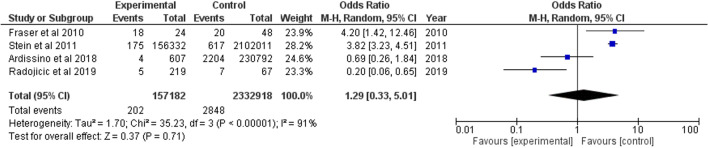
Forest plot showing odds of IIH in OSA patients

Four studies investigated OSA in IIH patients were included. One was a prospective cohort, one was case-control study, and the other two were database record studies. Overall pooled OR was estimated as 1.29 (95% CI 0.33, 5.01) (Fig. 8).

### Age-related macular degeneration

**Fig. 9:**
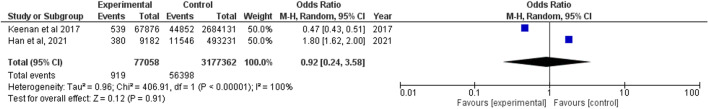
Forest plot showing odds of AMD in OSA patients

Two cohort studies reported incidence of OSA in an AMD population. A pooled OR of 0.92 [95% CI 0.24, 3.58] was concluded (Fig. 9).

## Discussion

The current systematic review and meta-analysis analyzed the incidence of OSA with common ocular disorders, and our analysis revealed significantly raised odds ratios for OSA with NAION, followed by FES, RVO, keratoconus, CSR, and glaucoma. No raised OR was observed with AMD. Aside from two studies, participants were either CPAP-naïve or the information was not disclosed. Therefore, the results of this study suggest people with OSA are at risk of serious eye disorders and would benefit from screening programs and follow-up to minimize their risk of irreversible vision loss and morbidity. Conversely, as OSA is an underdiagnosed disorder [59, 60], individuals presenting with ocular disorders should also be screened for OSA for holistic management.

This study found a 3.98 times greater risk for NAION with OSA, which is higher than a previous 2016 meta-analysis by Huon et al. where OSA conferred a 3.126 times greater risk for neuropathy [57]. NAION presents as a sudden, painless, usually unilateral, vision loss which commonly occurs upon awakening from sleep. It is thought to manifest from a microvascular infarction of the optic nerve from nocturnal hypoxemia of the short posterior ciliary arteries, which in this study shown is very likely to be precipitated by OSA [61]. Although the association of NAION and OSA has been discussed in various studies [11–20], the prevalence of OSA varies. Our meta-analysis included ten case-control studies where reported prevalence ranged from 0.06 to 94.73%. Of all studies, Bandi et al. [15] recorded the highest prevalence of NAION (18/19) OSA cases. The addition of Bandi et al., which was not in a previous meta-analysis, likely led to the higher OR reported by this study. The ranges of prevalence across studies may be due to heterogeneity in recruitment strategies for OSA, where Bandi et al. [15] used polysomnography and Li et al. [12] used the Sleep Apnea scale of the Sleep Disorders Questionnaire. Ethnic differences between Iranian and Caucasian populations also likely played a role in these differences. Even so, heterogeneity was high (*I*^2^ = 91%) and so NAION is likely raised as a product of OSA beyond a reasonable doubt.

Our meta-analysis showed OSA was associated with 3.68 times greater risk of developing FES which is slightly higher than a previous meta-analysis (OR = 3.126) [57]. FES is an ocular condition characterized by ease of eyelid eversion with slight upward traction and tissue that has become flaccid from loss of elasticity [62]. This meta-analysis identified prevalence ranges from 25.84% reported by Chambe et al. [8], to as high as 64.57% reported by Acar et al. [9] which upon further review, can be attributed to grading differences. While Chambe et al. [8] had split eyelid syndromes into FES and lax eyelid syndrome (LES), the latter being a milder version of the former, Acar et al. [9] graded FES. In considering LES, prevalence in OSA rose to 49.4% generally, and 75% in severe OSA, which ended up matching Acar’s finding of 74.6% for severe OSA. This likely unfortunately underestimates the OR in this analysis even though there was still a significantly raised odds ratio and reasonably low heterogeneity among the included studies (*I*^2^ = 29%).

Four studies were included in our analysis and the presence of OSA resulted in 2.71-fold increased odds of developing RVO, which was much higher Chou et al.’s reported OR of 1.94 [30, 57]. This is the first meta-analysis performed on RVO as the previous study had insufficient studies to analyze an association [57]. The risk of RVO may be increased by OSA through hypoxic insult to blood flow autoregulation mechanisms and the microvasculature, weakening the resilience of vessels to sudden occlusions. Considering the rise in risk with the addition of newer studies, more cohort studies should analyze this association to ensure consistency in risk and prevalence as RVO is an ophthalmic emergency, and elucidating risk in OSA patients can better educate practitioners.

KC, a non-inflammatory ectatic corneal condition characterized by progressive steepening and thinning of the cornea, was associated with higher risk of OSA in this study (OR = 1.87). It is suggested that higher activity of proteolytic enzyme matrix metalloproteinases (MMPs) degrade extracellular matrix in hypoxic response to stress or injury contributes to this association, as in KC patients MMP-9 in tears are increased and likely contribute to corneal thinning [63]. Our study included four case controls and a database study where the prevalence of keratoconus among OSA patients varied from 7.53% (11/146) reported by Gencer et al. [23] to 26.67% (4/15) reported by Pihlblad and Schaefer [22]. Our pooled OR estimate is close to that of the meta-analysis performed by Pellegrini M et al. [63] where the odds of OSA in KC patients was 1.8 times higher as compared to controls (OR = 1.841).

OSA has previously been associated with CSR, a condition of the eye where the neuro-sensory retina in the macular region is detached by the collection of serous fluid, resulting in mild loss of visual acuity, decreased contrast sensitivity, and visual and color distortion. Likely, increased circulating epinephrine and norepinephrine levels in OSA patients increase sympathetic tone, which causes endothelial dysfunction in the blood-retinal barrier and retinal serous fluid accumulation [64]. Our analysis included two studies with variance in prevalence ranging from 0.17 [29] to as high as 7.65% [28] and a pooled OR being 2.28. Our pooled estimate was almost in line with the study conducted by Wu et al. [65], which included six studies for systematic review and meta-analysis which showed CSR patients had 1.56 increased odds of having OSA than controls. This is considerably lower than the previous meta-analysis by Hou et al. although they only included two studies [26, 27].

Our meta-analysis included a total of 21 studies on the association between OSA and primary open-angle glaucoma, which was the largest study pool of all analyses. The meta-analysis showed 1.49 times higher risk for developing glaucoma in OSA, and variance in incidence ranged from 41.67% by Bilgin et al. [49] 41.67% (10/24), to 27.36% in a case-control study by Onen et al. [34] of 212 cases. By predisposing the optic nerve head to ischemia, OSA inflicts repeated hypoxic events, hemodynamic changes to retinal blood vessels, oxidative stress, mitochondrial dysregulation, and inflammation, which contribute to nerve fiber dysfunction and degeneration in glaucoma. It is possible the association of OSA with glaucoma may be partially confounded by other diseases resulting in poor perfusion, for example, obesity, hypertension, and diabetes [29].

While OSA has also been associated with AMD in the past [51], our pooled estimate from two included studies confers no raised odds of AMD in an OSA cohort. Recent research suggests hypoxia and oxidative stress from OSA trigger inflammatory processes which play roles in AMD pathogenesis [52]. In addition, OSA’s association with non-responsiveness to anti-VEGF for AMD treatments has perpetuated the belief it has some influence on the disease [51]; however, this meta-analysis proposes otherwise. While there are many overlapping factors that may hinder a true association, including obesity and older age [52], it should be considered only two studies were available for analysis. Therefore, future research should investigate the certainty of our findings further.

This study collectively examined associations between OSA and a range of ocular conditions and analyzed the association with IIH for the first time. Overall pooled OR from four included studies was 1.29, suggesting slightly raised odds of sleep apnoea with IIH (*p* < 0.001). OSA is known to increase intracranial pressure through hypercapnia, hypoxia, and cerebral vasodilation that increases intracranial volume [14]. A paper has suggested use of CPAP can reverse IIH in those with contiguous OSA, supporting this link and providing a realistic solution if IIH is coincident [14]. As some studies did not control obesity, it is possible this is a confounder for this finding considering body weight can limit chest expansion and cause hypoxia and hypercapnia like OSA [14].

Our findings highlight OSA is associated with a raised OR for a range of serious and debilitating eye disorders, and an increase in studies available for meta-analysis has allowed for these relationships to be better elucidated. Considering retinal tissue has one of the highest oxygen demands in the body, it is clearly liable to hypoxic injury by nocturnal events in OSA patients [29]. Thus, regular screening of OSA patients for ophthalmologic complications should be encouraged to protect patients from irreversible vision loss.

Despite these contributions, some limitations should be addressed. First, meta-regression analysis of various confounding factors was beyond the scope of this study due to age, sex, ethnicity, and disease duration that can deter the odds ratio. It did not help that confounding variables like hypertension, diabetes, body mass index, and other systemic diseases prevalent in both OSA and eye diseases were not reported by studies in OSA participants with eye diseases. Despite this, it was observed most studies had multivariate cox regression models, propensity-matched participants, and backward stepwise analyses in place to minimize confounding, even if these were not explicitly listed as characteristics of OSA participants with eye diseases. Future studies could be improved by ensuring multivariate models were in place across all studies to ensure a finding is independent of other confounders. Second, the severity of OSA in various patients was not often evaluated, which could have otherwise affected the odds of ocular morbidities, and CPAP treatment status was not explicitly listed in most studies. While it was clear in prospective studies that participants were CPAP-naïve, retrospective studies using ICD codes did not detail whether participants were offered/on therapy, making it possible endothelial dysfunction caused by OSA could be reversed and minimized as a cause for ocular disease. Even so, OSA preceded the onset of eye diseases in these studies, and retrospective study designs were most likely to rigorously control for confounding factors. This makes it likely OSA contributed to the event nonetheless, although the effect may be augmented if some populations were treated with CPAP. Third, the vast variance in prevalence of our pooled studies is due to heterogeneity of study designs between studies and may impact generalizability. Because the database studies had an enormous sample size to pool estimates while case-control studies had limited samples in comparison, this likely exacerbates the effect. Also, the sample size for some ocular morbidities varies in comparison to others due to varied prevalence rates.

## Conclusion

This systematic review and meta-analysis study showed significant associations between OSA and NAION, FES, RVO, KC, CSR, and glaucoma, but no association with AMD. Considerable heterogeneity throughout analyses may impact the generalizability of our findings. Vision specialists and referring physicians should be aware of the risks associated with OSA, and various ocular morbidities and patients referred for early recognition, diagnosis, and treatment. Conversely, ophthalmologists seeing patients with any of these conditions should consider referring patients for assessment of possible OSA if that has not already occurred.
